# *Phyllanthus* Niruri L. Exerts Protective Effects Against the Calcium Oxalate-Induced Renal Injury *via* Ellgic Acid

**DOI:** 10.3389/fphar.2022.891788

**Published:** 2022-06-16

**Authors:** Mao-Ting Li, Lu-Lu Liu, Qi Zhou, Lin-Xi Huang, Yu-Xuan Shi, Jie-Bin Hou, Hong-Tao Lu, Bing Yu, Wei Chen, Zhi-Yong Guo

**Affiliations:** ^1^ Changhai Hospital, Naval Medical University, Shanghai, China; ^2^ Department of Nephrology, the Second Medical Centre, Chinese PLA General Hospital, Beijing, China; ^3^ Department of Naval Medicine, Naval Medical University, Shanghai, China; ^4^ Department of Cell Biology, Center for Stem Cell and Medicine, Navy Medical University, Shanghai, China

**Keywords:** *Phyllanthus* niruri L., calcium oxalate-induced renal injury, network pharmacology, ellagic acid, lipid nephrotoxicity

## Abstract

**Background:** Urolithiasis or kidney stones is a common and frequently occurring renal disease; calcium oxalate (CaOx) crystals are responsible for 80% of urolithiasis cases. *Phyllanthus* niruri L. (PN) has been used to treat urolithiasis. This study aimed to determine the potential protective effects and molecular mechanism of PN on calcium oxalate-induced renal injury.

**Methods:** Microarray data sets were generated from the calcium oxalate-induced renal injury model of HK-2 cells and potential disease-related targets were identified. Network pharmacology was employed to identify drug-related targets of PN and construct the active ingredient-target network. Finally, the putative therapeutic targets and active ingredients of PN were verified *in vitro* and *in vivo*.

**Results:** A total of 20 active ingredients in PN, 2,428 drug-related targets, and 127 disease-related targets were identified. According to network pharmacology analysis, HMGCS1, SQLE, and SCD were identified as predicted therapeutic target and ellagic acid (EA) was identified as the active ingredient by molecular docking analysis. The increased expression of SQLE, SCD, and HMGCS1 due to calcium oxalate-induced renal injury in HK-2 cells was found to be significantly inhibited by EA. Immunohistochemical in mice also showed that the levels of SQLE, SCD, and HMGCS1 were remarkably restored after EA treatment.

**Conclusion:** EA is the active ingredient in PN responsible for its protective effects against CaOx-induced renal injury. SQLE, SCD, and HMGCS1 are putative therapeutic targets of EA.

## 1 Introduction

*Phyllanthus* niruri L. (PN) belongs to *Phyllanthus* Linn., Subgen. *Phyllanthus*, Sect. *Phyllanthus* and the species *Phyllanthus* niruri. It is distributed in China, India, Indochina Peninsula, Malay Archipelago, and tropical America (China Plants Database, http://db.kib.ac.cn/CNFlora/HierarchicalSearch.aspx). Since the 1940s, the chemical constituents of PN have been systematically studied, and the plant has been reported to contain a variety of beneficial ingredients (Krishnamurti and Seshadri, 1946). Pharmacological studies have shown that it has anti-cancer, anti-inflammatory, anti-oxidation, anti-fungal, anti-virus, and other activities. PN as a diuretic is widely used in clinical medicine, as a traditional treatment in Brazil (Kaur et al., 2017; Kieley et al., 2008). In addition, A.H.Campos et al. (Freitas et al., 2002) and J.L.Nishiura et al. (Nishiura et al., 2004) have shown via experimental and clinical studies, respectively, that PN can treat kidney stones.

Urolithiasis or kidney stones is a common and frequently occurring disease worldwide. It is mainly characterized by back pain, abdominal pain, hematuria, nausea, and vomiting. It can cause serious urinary tract infection, acute renal function declines, urinary tract obstruction, and other adverse consequences. The prevalence of urolithiasis in China is 7.54%, and the recurrence rate is 50% 5–10 years after the first treatment (Wang et al., 2017). Stone formation in urolithiasis occurs due to the accumulation of human metabolites, therefore, some patients are closely related to metabolic factors. Metabolic abnormalities, such as hypercalciuria, hyperoxaluria, and hyperuricemia, occur in a high proportion of patients with kidney stones (Abu-Ghanem et al., 2016). Calcium oxalate (CaOx) crystals are implicated in 80% of urolithiasis cases (Evan, 2010; Khan et al., 2016).

Renal tubular epithelial cells are the primary targets of CaOx-induced renal injury. CaOx crystals interact with renal tubular epithelial cells, resulting in cellular injury that becomes the attachment site of crystals (Scheid et al., 2004). The damage of renal tubular epithelial cells leads to further crystallization, crystal retention, and development of stone (Khan, 2004). It is worth noting that during this process, renal tubular cells undergo some adaptive changes to prevent subsequent harm. This reaction is termed acquired renal cytoresistance, which is characterized by the accumulation of cholesterol in cells (Oner and Cirrik, 2009). Cholesterol accumulation increases plasma membrane stability and protects cells from further toxic damage (Zager et al., 1999). However, if left unchecked, these initially beneficial effects may increase the risk of kidney stones (Taguchi et al., 2020; Wang et al., 2022) and progressive renal injury (Kim et al., 2009; Zager et al., 2011). In addition, it is reported that renal tubular cell injury is mediated by lipid accumulation associated with changes in gene expression related to cholesterol transport and synthesis (Kim et al., 2020). In this study, we investigated the mechanisms by which PN manifests its protective effects against CaOx-induced renal injury for a better understanding of its protective properties, especially those related to lipid nephrotoxicity.

## 2 Materials and Methods

### 2.1 Cell Culture and Treatment

Human renal tubular epithelial HK-2 cells were purchased from American Type Culture Collection (ATCC; Manassas, United States). The cells were cultured in Dulbecco’s Modified Eagle Medium/Nutrient Mixture F-12 (DMEM/F-12; Hyclone, United States), containing 10% fetal bovine serum (Gibco, United States), 100 U/mL penicillin, and 100 U/mL streptomycin (Sangon, China). Cells were cultured at 37°C and 5% CO2. The cells were divided into the following three groups: 1) cells in the sodium oxalate (NaOx; Su Yi Chemical Reagent Co., Ltd., Shanghai, China) group were incubated in DMEM/F12 at a concentration of 1 mM (NaOx group) for 12 or 24 h, 2) cells in the Ellagic acid (NaOx + EA group) were incubated in DMEM/F12 for 12 or 24 h, 3) cells in the PBS were incubated in DMEM/F12 (Ctrl group). The EA concentration was selected based on the results of a Cell Counting Kit 8 (CCK8) assay (Beyotime, China). EA was purchased from Source Leaf Creature (Shanghai, China).

### 2.2 Microarray Analysis

The duration (24 h) and concentration (1 mM) of NaOx exposure of the cell model were set based on the results of a previous study (Huang et al., 2005). Microarray analysis was performed using Affymetrix HTA 2.0 Transcriptome Arrays.

### 2.3 Network Pharmacology

The active ingredients in PN were determined from available data reported in the literature and the potential drug-related targets were determined from the STITCH database (confidence score > 0.15, http://stitch.embl.de/) and PharmMapper Server (The top 100 pharmacophore candidates, http://www.lilab-ecust.cn/pharmmapper/). The disease-related targets were screened from microarray analysis and the conditions were set as FC ≥ 2 and *p* value < 0.05 or FC ≤ 0.5 and *p* value < 0.05. Next, the targets of PN active ingredients were mapped to disease-related targets to obtain the predicted therapeutic targets. The active ingredient-target network was constructed using Cytoscape (version 3.8.0) to comprehensively understand the complex interactions between PN, its active ingredients, and their therapeutic targets. The main active ingredients and their putative therapeutic targets were determined.

### 2.4 Molecular Docking

Molecular docking was performed using AutoDock Vina (version 1.1.2, US) (Trott and Olson, 2010). The simplified molecular input line entry system (SMILES) structure of EA (ingredient CID: 5,281,855) was obtained from PubChem (https://pubchem.ncbi.nlm.nih.gov/). The interactions between EA and its putative therapeutic targets were predicted by AutoDock Vina. The docking scores (to predict binding affinity) and the EA-protein complexes were extracted from AutoDock Vina. Ligand-protein interactions were analyzed using LigPlus (version 2.24, UK) (Laskowski and Swindells, 2011) software and two-dimensional figures were obtained. Then, the PyMoL (version 4.5.0, https://www.schrodinger.com/pymol) is used to make three-dimensional figures.

### 2.5 Animals

Male C57BL/6 mice aged 6 weeks were (Changzhou Cavens Lab Animal Co., Ltd. Jiangsu, China) maintained in a special pathogen-free (SPF) animal house at 25°C with 12 h of light per day and free access to water and food. All animal procedures are approved by the Laboratory Animal Ethics Committee of Naval Medical University. The mice (*n* = 18) were randomly divided into the following three groups: control group (Con; *n* = 6), glyoxylate-induced CaOx group (Gly; *n* = 6), and EA treatment group (Gly + EA; *n* = 6). The Gly and Gly + EA group mice were subjected to intraperitoneal injection of glyoxylate (80 mg/kg; UDChem Technology Co., Ltd. Shanghai, China) once daily for 7 days and the Con group was treated with the same volume of saline. The Gly + EA group was intragastrically administrated once daily with EA (20 mg/kg) for 7 days. On the eighth day, all mice were sacrificed; their left kidney tissues were collected and fixed in 10% formaldehyde, embedded in paraffin, cut into 4 μm sections, subjected to hematoxylin and eosin (HE) staining and immunohistochemical analysis; after the right kidney was homogenized, the relative calcium content of total protein, GPx activities, and MDA content were determined by calcium assay kit, GPx, and MDA detection kits, respectively. (Jiancheng, Nanjing, China). The blood was collected and centrifuged to extract the serum for serum creatinine detection.

### 2.6 Western Blot

Total protein was isolated from HK-2 cells. The HK-2 cell proteins samples were incubated with anti-GAPDH (1:1,000, Proteintech), anti-β-actin (1:1,000, Proteintech), anti-SCD (1:1,000, Proteintech), anti-SQLE (1:1,000, Proteintect), anti-HMGCS1(1:1,000, Proteintech) and anti-p53 (1:1,000, Proteintech) overnight. The secondary antibodies (Licor, United States) were incubated at room temperature for 2 h. The intensity of the immunofluorescent signals was detected using the Odyssey fluorescence imaging system (GENE, United States).

### 2.7 Histology, Immunohistochemistry (IHC) and Immunofluorescence

Mouse kidneys were fixed in 10% formalin, embedded in paraffin, cut into 4 μm-thick sagittal sections, and stained with HE. Treated HK-2 cells were fixed with 4% paraformaldehyde, permeabilized with 1% Triton X-100, and then blocked with BSA. Both HK-2 cells and kidney tissue sections were incubated overnight with anti-SCD (1:1,000, Proteintech), anti-SQLE (1:1,000, Proteintech), and anti-HMGCS1 (1:1,000, Proteintech) antibodies. The cell nuclei were stained with DAPI after incubating with Alexa Fluor 488 (1:100) for 1 h in the dark. The fluorescence intensity of the HK-2 cells was observed under a confocal microscope. Representative IHC images of kidney sections (200x magnification) were selected and semiquantitative analysis of images using ImageJ software (version 1.6.0, US) are displayed.

### 2.8 Statistical Analysis

The experimental data are expressed as (mean ± standard error). The SPSS (version 19.0, US) software was used for statistical data analysis. Graphs were made using GraphPad Prism (version 9.0, US). The independent *t*-test and one-way ANOVA analysis were used for analyzing the difference between experimental groups. A difference of *p* < 0.05 between the groups was considered statistically significant.

## 3 Results

### 3.1 Network Pharmacology Analyses

#### 3.1.1 Active Ingredient Screening

The active ingredients in PN were determined from literature (Bagalkotkar et al., 2006; Li et al., 2007; Kaur et al., 2017; Jantan et al., 2019). The active ingredients that were discussed in at least 75% of the literature were shortlisted for our analysis. As described in Table 1 active ingredients of PN were considered for our analysis.

**TABLE 1:** List of the 20 active ingredients of *Phyllanthus* niruri L.

No.	Chemical composition	References
1	Quercitrin	Bagalkotkar et al. (2006); Li XR et al. (2007); Kaur et al. (2017); Jantan et al. (2019)
2	Rutin	Bagalkotkar et al. (2006); Li XR et al. (2007); Kaur et al. (2017); Jantan et al. (2019)
3	Niranthin	Bagalkotkar et al. (2006); Li XR et al. (2007); Kaur et al. (2017); Jantan et al. (2019)
4	Nirtetralin	Bagalkotkar et al. (2006); Li XR et al. (2007); Kaur et al. (2017); Jantan et al. (2019)
5	Phyllanthin	Bagalkotkar et al. (2006); Li XR et al. (2007); Kaur et al. (2017); Jantan et al. (2019)
6	Phyltetralin	Bagalkotkar et al. (2006); Li XR et al. (2007); Kaur et al. (2017); Jantan et al. (2019)
7	Corilagin	Bagalkotkar et al. (2006); Li XR et al. (2007); Kaur et al. (2017); Jantan et al. (2019)
8	Ellagic acid	Bagalkotkar et al. (2006); Li XR et al. (2007); Kaur et al. (2017); Jantan et al. (2019)
9	Astragalin	Bagalkotkar et al. (2006); Li XR et al. (2007); Kaur et al. (2017); Jantan et al. (2019)
10	Quercetin	Bagalkotkar et al. (2006); Kaur et al. (2017); Jantan et al. (2019)
11	Lupeol	Bagalkotkar et al. (2006); Kaur et al. (2017); Jantan et al. (2019)
12	Cubebin dimethyl ether	Bagalkotkar et al. (2006); Kaur et al. (2017); Jantan et al. (2019)
13	Urinatetralin	Bagalkotkar et al. (2006); Li XR et al. (2007); Kaur et al. (2017)
14	Hinokinin	Li XR et al. (2007); Kaur et al. (2017); Jantan et al. (2019)
15	Hypophyllanthin	Bagalkotkar et al. (2006); Kaur et al. (2017); Jantan et al. (2019)
16	Isolintetralin	Bagalkotkar et al. (2006); Li XR et al. (2007); Jantan et al. (2019)
17	Lintetralin	Bagalkotkar et al. (2006); Li XR et al. (2007); Kaur et al. (2017)
18	Phyllnirurin	Bagalkotkar et al. (2006); Li XR et al. (2007); Kaur et al. (2017)
19	Phyllanthine	Bagalkotkar et al. (2006); Li XR et al. (2007); Kaur et al. (2017)
20	Gallic acid	Bagalkotkar et al. (2006); Kaur et al. (2017); Jantan et al. (2019)

#### 3.1.2 The Active Ingredient-Predicted Therapeutic Target Network Analysis

**FIGURE 1:**
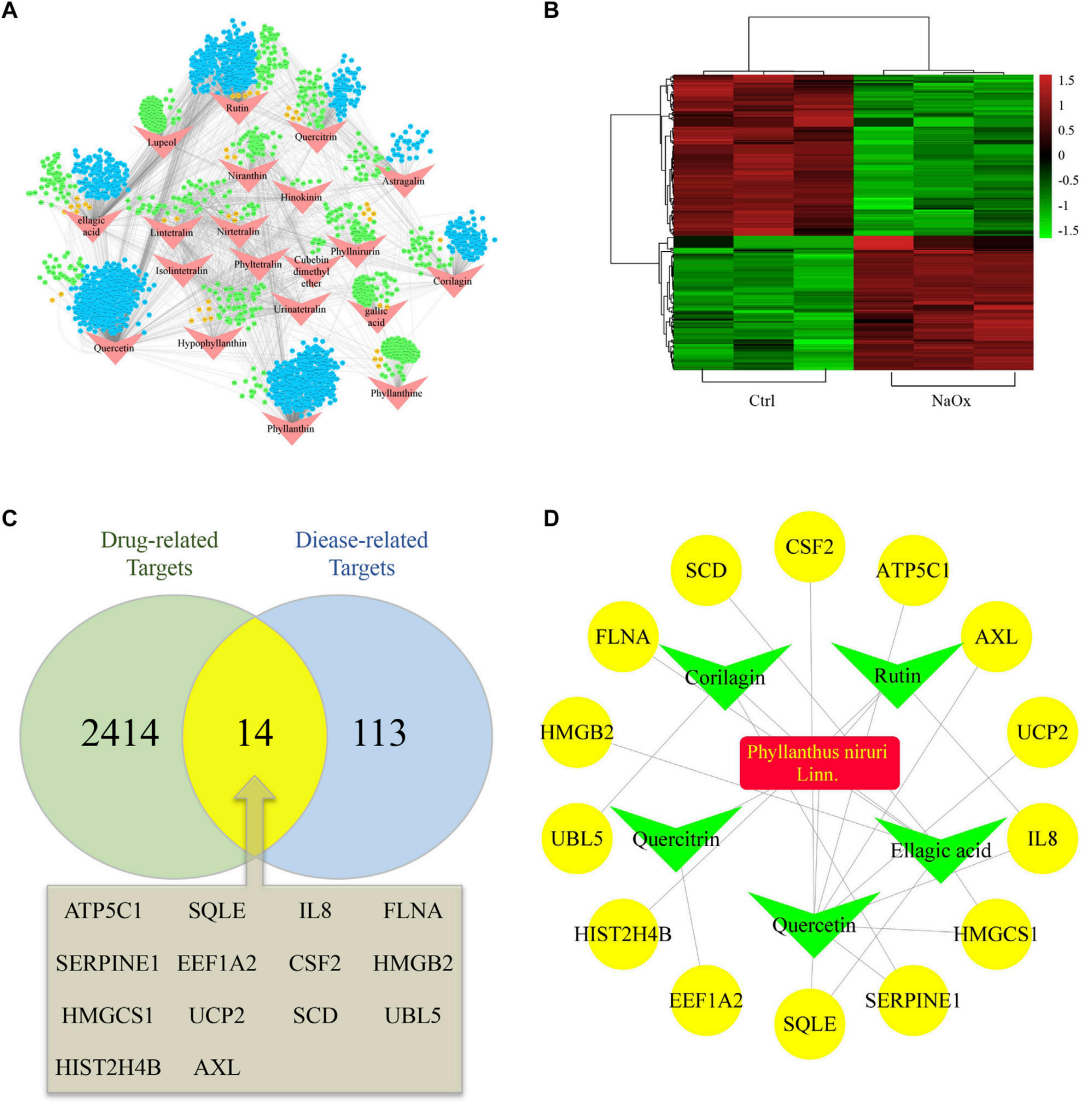
Hub ingredients and targets screening **(A)** An ingredient-target network and nodes represent ingredients (red) and targets form STITCH (blue), Pharmmapper (green) or both (orange) **(B)** Heatmap of potential disease-related targets on microarray analysis between HK-2 cells cultured with (NaOx group) or without (Ctrl group) NaOx **(C)** Venn diagram showing the overlap of diease-related (Oxalate-induced renal injury related) targets (blue) and drug-related (*Phyllanthus *niruri. L related) targets (green) **(D)** The active ingredient-predicted therapeutic target network.

The STITCH database and PharmMapper Serve were used together to screen the targets of 20 active ingredients of PN. Totally, 1,688 and 781 potential targets were retrieved from the above two databases, respectively (Supplementary Tables S1,2). After removing the repetitive targets, the total amount of obtained potential drug-related targets reduced to 2,428 (Figure 1A). In order to probe the potential disease-related targets, we performed transcription profiling on affymetrix microarray HTA 2.0. Figure Figure1B shows the differential expression of the 127 targets (Supplementary Table S3).

At the intersection of potential drug-related targets and disease-related targets were 14 potential therapeutic targets, as shown by the venn diagram (Figure 1C). Inputting this information, an active ingredient-predicted therapeutic target network was visualized using Cytoscape. As shown in Figures 1A,D total of 23 relationships (edges) between 20 nodes were identified. Degree of active ingredients analyzed by NetworkAnalyzer tool in Cytoscape (Supplementary Table S4), and the top two hub active ingredients, quercetin, and EA were identified. Of interest is that EA has important antioxidant, anti-inflammatory, and anti-apoptotic effects, and has been shown to improve kidney histology and decrease kidney injury biomarker levels (Liu et al., 2020; Neamatallah et al., 2020). In addition, the hub targets corresponding to EA were SQLE, HMGCS1, SCD, HMGB2, and FLNA. Of these, SQLE, HMGCS1, and SCD are related to lipid metabolism and cholesterol synthesis (Brown and Goldstein, 1997; Samuel et al., 2014).

#### 3.1.3 Molecular Modeling and Ligand Docking

**FIGURE 2:**
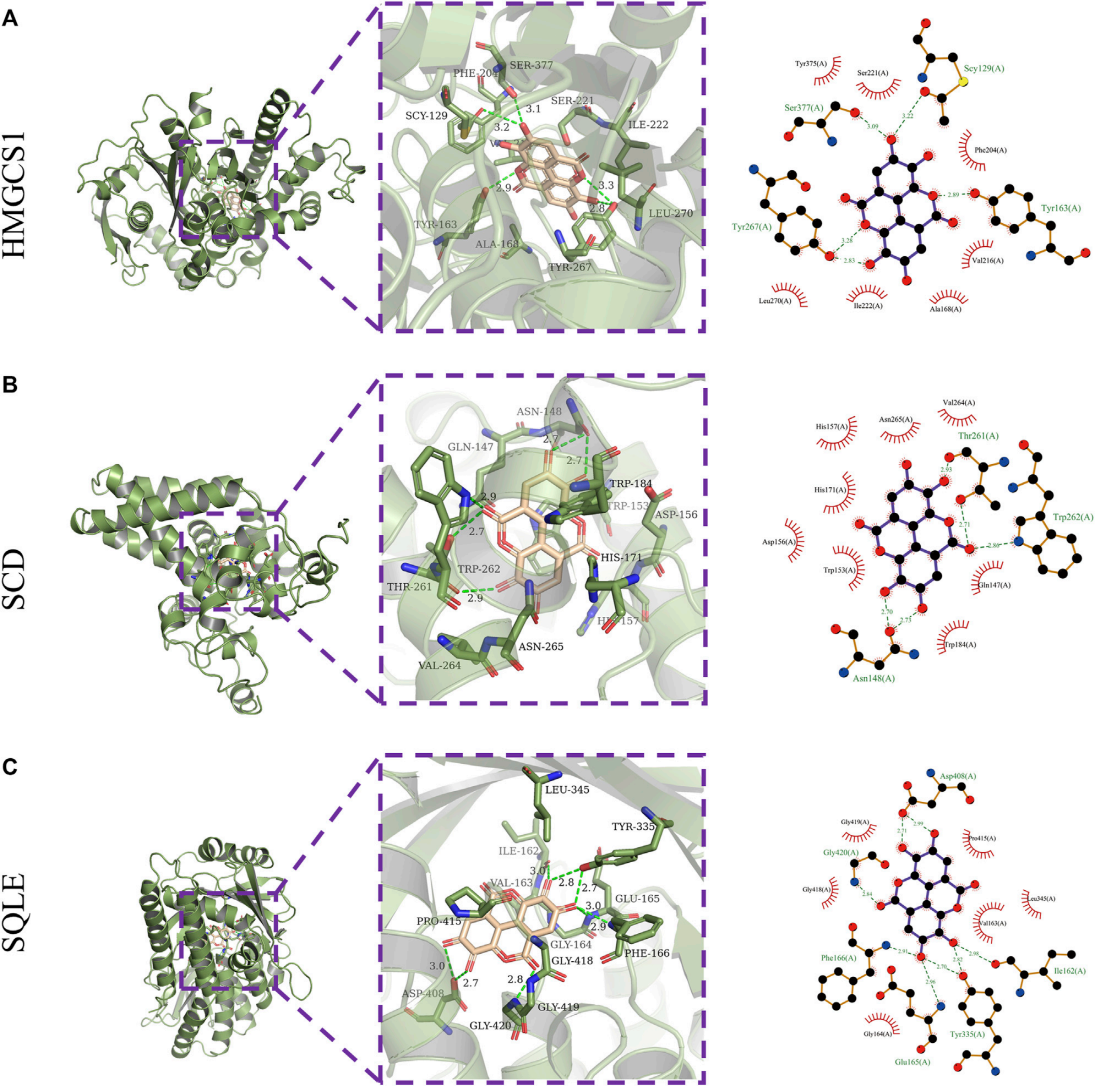
Molecular docking. Three-dimensional (left) and two-dimensional (right) ligand interaction diagrams of ellagic acid and the original ligands of HMGCS1 **(A)**, SCD **(B)**, and SQLE **(C)**.

Next, the binding affinities between EA and the three therapeutic targets, SQLE, SCD, and HMGCS1, were evaluated using the AutoDock Vina software. Molecular docking programs use scoring functions to evaluate the binding energy of predicting ligand-receptor complexes. As shown in Table 2, the scores of the binding energies of EA with SCD, SQLE, and HMGCS1 are −10.6 kcal/mol, −9.8 kcal/mol, and −7.6 kcal/mol, respectively. The two-dimensional and three-dimensional molecular docking diagrams of the three therapeutic targets with EA and its original ligands are shown in Figure 2 and Table 2. All of them have low docked binding energy, which is considered desirable.

**TABLE 2:** The AutoDock Score of putative targets with ellagic acid and the amino acid residue of targets via hydrogen bonds and hydrophobic contact.

Gene	PDB accession number	Ligand ID	Autodock score (kcal/mol)	Hydrophilic interactions	Hydrophobic contacts
SCD	6C6R	FAD	−10.6	Thr261(A), Trp262(A), Asn148(A)	His157(A), Asn265(A), Val264(A), His171(A), Asp156(A), Trp153(A), Trp184(A), Gln147(A)
SQLE	4ZYO	ST9	−9.8	Asp408(A),Gly420(A), Phe166(A),Glu165(A), Tyr335(A), Ile162(A)	Gly419(A), Gly418(A), Gly164(A), Val163(A), Leu345(A), Pro415(A)
HMGCS1	2P8U	COA	−7.6	Scy129(A), Tyr163(A), Tyr267(A), Ser377(A)	Tyr375(A), Ser221(A), Phe204(A), Val216(A), Ala168(A), Ile222(A), Leu270(A)

### 3.2 SQLE, HMGCS1 and SCD Expression in the Model of Oxalate Renal Injury

**FIGURE 3:**
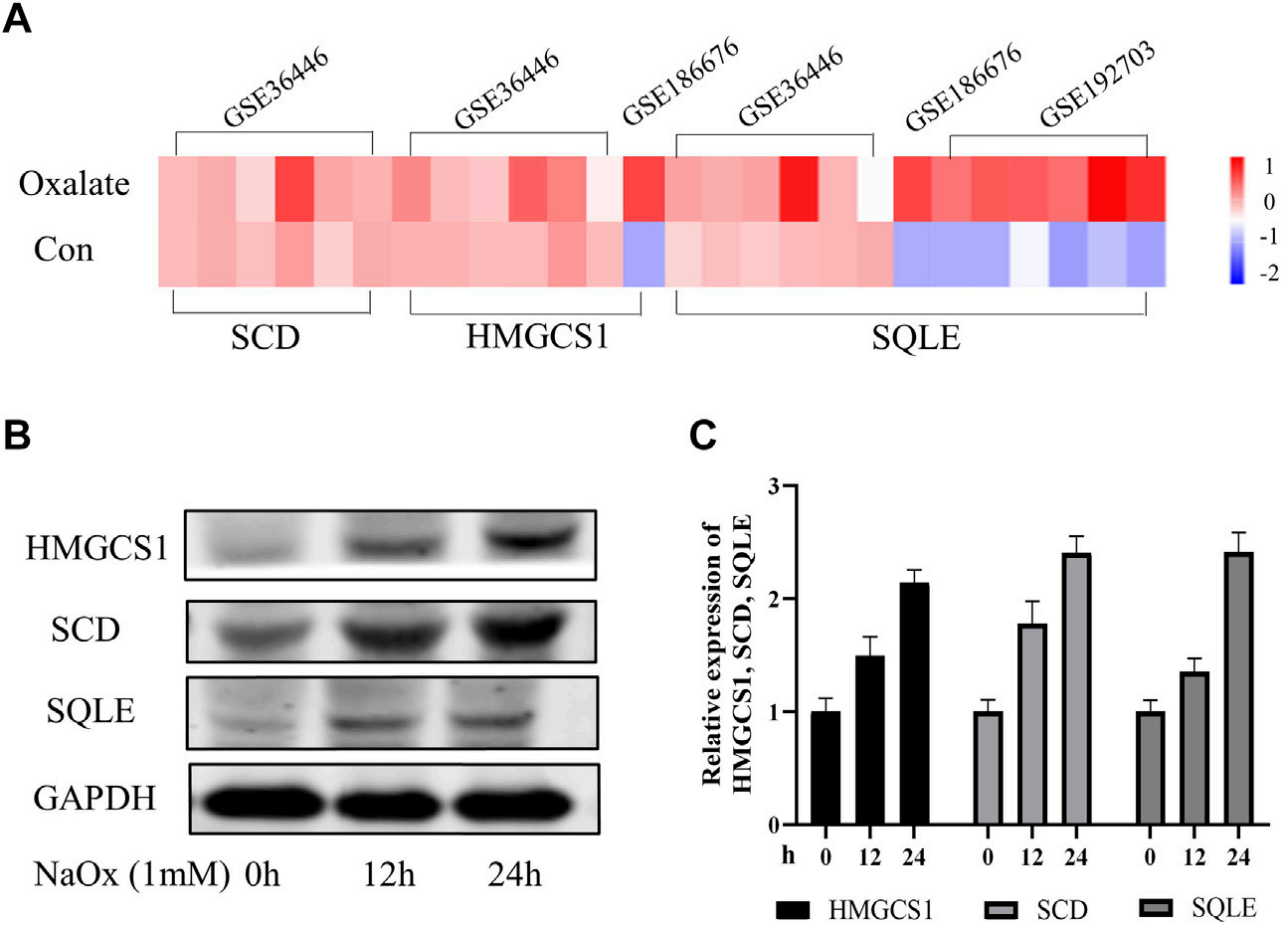
The changing trend of SQLE, HMGCS1, and SCD in oxalate-induced renal injury models **(A)** The Heatmap of SQLE, HMGCS1and SCD were shown in three GSE databases **(B)** Expression of SQLE, HMGCS1 and SCD protein was detected by Western blot in the sodium oxalate group (NaOx) at 0, 12, and 24 h **(C)** Western blot displayed as column charts after quantification.

In order to further explore the expression characteristics of SQLE, HMGCS1 and SCD in oxalate renal injury models, we initially screened for potential SQLE, HMGCS1 and SCD by determining transcripts in three data sets (GSE36446, GSE186676, and GSE192703). The GSE datasets were downloaded from the GEO database (https://www.ncbi.nlm.nih.gov/geo/). The results showed that SQLE was up-regulated in mice model of glyoxylate (Gly)-induced oxalate renal injury; SQLE and HMGCS1 were up-regulated in HEK293 T cells model of calcium oxalate monohydrate (COM)-induced oxalate renal injury; SQLE, HMGCS1and SCD were up-regulated in rats model of hydroxy-l-proline (HLP)-induced oxalate renal injury (Figure 3A). At the same time, our HK-2 cell model experiment *in vitro* verified that the expressions of SQLE, SCD, and HMGCS1 increased with the increasing time of NaOx stimulation (Figures 3B,C, Supplementary Table S5). The same trend showed that SCD, HMGCS1 and SQLE were up-regulated in oxalate-induced renal injury.

### 3.3 Ellagic Acid Protects HK-2 Cells Against Oxalate-Induced Injury by Reducing the Expression of SQLE, HMGCS1, and SCD

**FIGURE 4:**
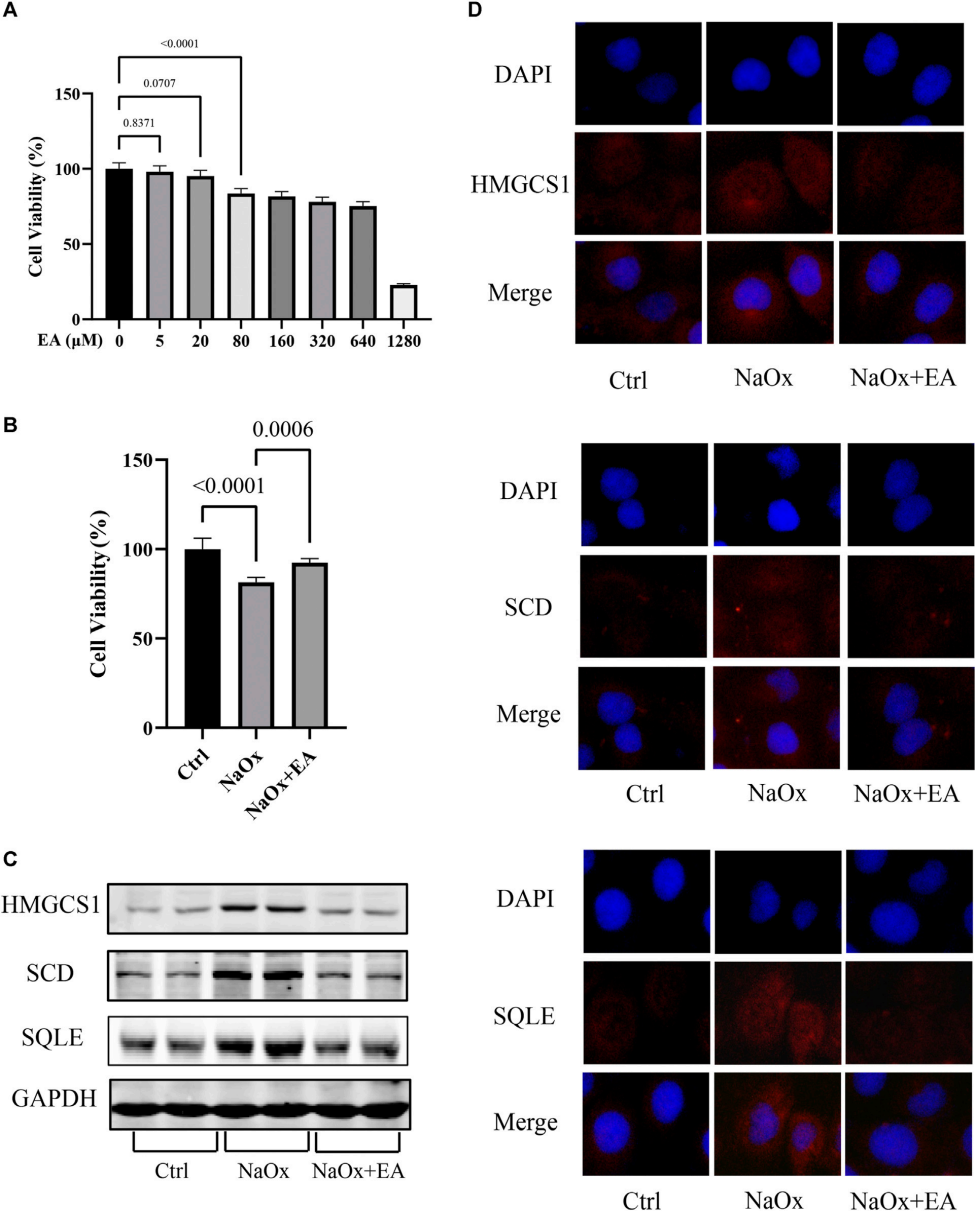
Ellagic acid protects NaOx-induced injury in cells and reduces the expression of SQLE, HMGCS1, and SCD **(A)** The cytotoxicity of ellagic acid on HK-2 cells determined by the CCK8 assay **(B)** The cytotoxicity of ellagic acid on NaOx treated HK-2 cells determined by the CCK8 assay **(C)** The expression of SQLE, HMGCS1, and SCD by Western blot in the control group (Ctrl), sodium oxalate group (NaOx), and ellagic acid treatment group (NaOx + EA) **(D)** Cells were analyzed by immunocytochemistry (100X) in the control group (Ctrl), sodium oxalate group (NaOx), and ellagic acid treatment group (NaOx + EA).

The cytotoxic effect of EA was investigated using HK-2 cells with CCK8 assay. As shown in Figure 4A, we chose a concentration of 20 µM of EA for the experiments (Supplementary Table S6). Furthermore, The cellular viability of HK-2 cells was inhibited after NaOx stimulation, but restored after treatment with EA (Figure 4B, Supplementary Table S7). The western blot analysis showed that EA treatment downregulated NaOx-induced injury elevation of SQLE, HMGCS1, and SCD (Figure 4C). As shown in Figure 4D, the fluorescence intensity of SQLE, HMGCS1, and SCD in the NaOx group was higher than that in the ctrl group, and the fluorescence intensity decreased after EA treatment.

### 3.4 Ellagic Acid Protects Calcium Oxalate-Induced Renal Injury in Mice by Reducing the Expression of SQLE, HMGCS1, and SCD

**FIGURE 5:**
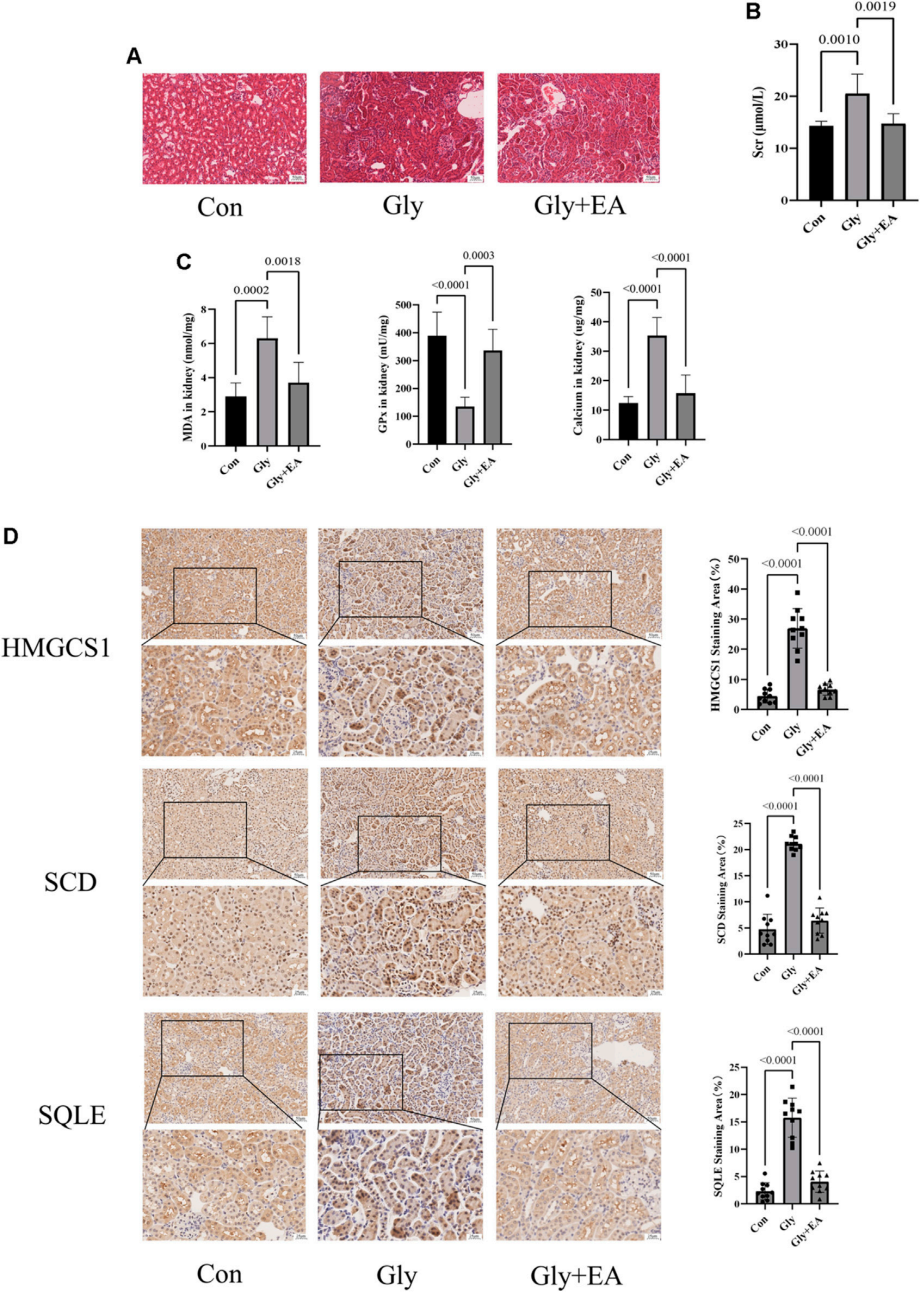
Ellagic acid protects calcium oxalate-induced renal injury in mice and reduces the expression of SQLE, HMGCS1, and SCD **(A)** Representative light microscopy images of hematoxylin and eosin staining of kidneys from the control group (Con), glyoxylate-induced CaOx group (Gly), and ellagic acid treatment group (Gly + EA) (magnification, ×200; scale bar = 50 μm) **(B)** Serum creatinine level in control group (Con), glyoxylate-induced CaOx group (Gly), and ellagic acid treatment group (Gly + EA) **(C)** The GPx activities, MDA, and total calcium content in control group (Con), glyoxylate-induced CaOx group (Gly), and ellagic acid treatment group (Gly + EA) **(D)** Expression of SCD, HMGCS1, and SQLE were analyzed by immunohistochemistry (IHC) (left) Representative light microscopy images of IHC staining of kidney of mice (magnification, ×200; scale bar = 50 μm (up) and 25 μm (down)); (right) Semi-quantitative score of SCD, HMGCS1, and SQLE.

As shown in Figures 5A,B, EA protects CaOx-induced renal injury in mice. In the HE staining of kidney sections, compared with the Con group, interstitial cell infiltration in Gly mice was significantly more severe. After EA intervention, the injury of renal tubules was gradually alleviated. Serum creatinine level was significantly decreased after EA intervention (Supplementary Table S8). In addition, the oxidative stress in mice kidney was evaluated by determining glutathione peroxidase (GPx) activities and malondialdehyde (MDA) content. As shown in Figure 5C, MDA content in the Gly mice increased significantly compared with that in the Con mice, and GPx activities in Gly mice decreased significantly compared with those in the Con mice. All changes after EA intervention can be called back. From the total calcium content in the kidney of different groups of mice (Figure 5C), it can be seen that EA intervention can significantly reduce the total calcium content of Gly mice. The IHC result was shown in Figure 5D, the expression of SCD HMGCS1 and SQLE in the Gly group was considerable increased compared with that in the Con group. After EA treatment, the expression of these indicators decreased. This finding was further confirmed by IHC semiquantitative analysis.

### 3.5 Ellagic Acid Protects HK-2 Cells Against Oxalate-Induced Injury by Reducing the Expression of p53

**FIGURE 6:**
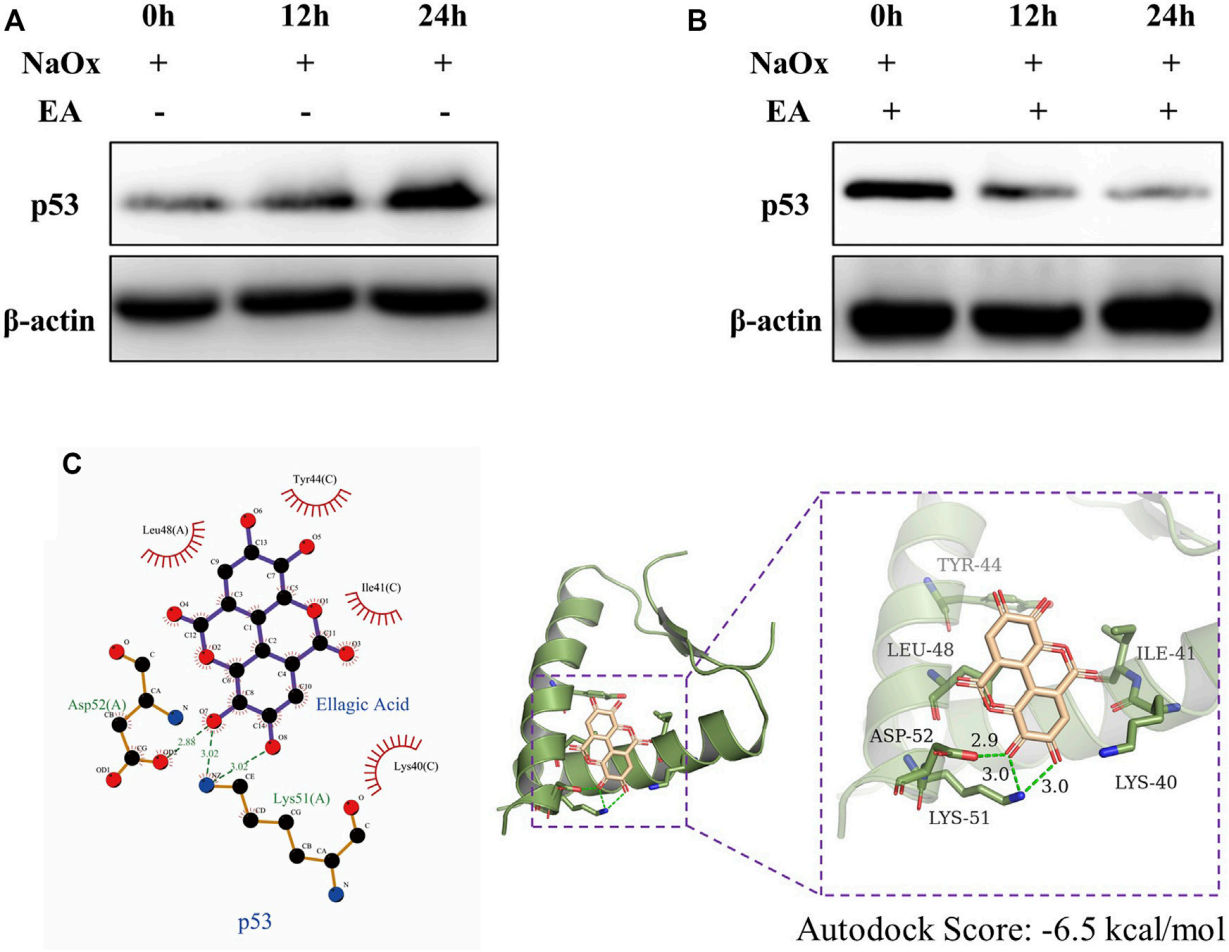
Ellagic acid reduces the expression of p53 **(A)** Expression of p53 protein was detected by western blot in the sodium oxalate group (NaOx) at 0, 12, and 24 h **(B)** Expression of p53 protein was detected by Western blot in the ellagic acid treatment group (NaOx + EA) at 0, 12, and 24 h **(C)** Two-dimensional (left) and three-dimensional (right) ligand interaction diagrams of ellagic acid and the original ligands of p53.

We used the PROMO database to predict the common transcription factors of SCD, SQLE, HMGCS1, which also included p53 (Supplementary Figure S1). As shown in Figure 6A, in our HK-2 cell model experiment *in vitro*, the western blot analysis showed that the expressions of p53 increased with the increasing time of NaOx stimulation. The change can be reversed by the addition of EA (Figure 6B). In addition, the results of molecular docking show that the two-dimensional and three-dimensional molecular docking diagrams of p53 with EA (Figure 6C). The score of the binding energies is -6.5 kcal/mol.

## 4 Discussion

Natural herbal medicines are typical multi-component, multitarget, and multi-pathway agents; they contain active ingredients which are responsible for their pharmacological activity. Our study aimed to identify the key active ingredients of PN, which has several medicinal properties, and their putative therapeutic targets based on network pharmacology.

Network pharmacology is a promising approach for the study of traditional Chinese medicine (TCM). In recent years, with the popularization of network pharmacology, an integrated approach of network pharmacology and multi-omics has become an important tool for analyzing the mechanisms of action of TCM. Transcriptomics has been widely applied with network pharmacology analysis to characterize the molecular mechanisms underlying therapeutic effects. In this study, we employed network pharmacology and transcriptomics to analyze the mechanism of PN in the treatment of CaOx-induced renal injury and enabled the identification of the active ingredient (EA) in PN and its core targets (HMGCS1, SQLE, and SCD). Moreover, the experimental results were consistent with the results of network pharmacology mining, increasing the reliability of network pharmacology network prediction.

3-hydroxy-3-methylglutaryl-CoA synthase 1 (HMGCS1) is a metabolic enzyme involved in the formation of 3-hydroxy-3-methylglutaryl-CoA (HMG-CoA), an important substrate in mevalonate pathway (Gruenbacher and Thurnher, 2015). The mevalonate pathway is an enzymatic cascade responsible for synthesizing cholesterol (Thurnher et al., 2013). Squalene epoxidase (SQLE) is one of the rate-limiting enzymes in the biosynthesis of cholesterol. SQLE can further affect the synthesis of cholesterol in mevalonate pathway by affecting the catalysis of squalene (Yu et al., 2020). Stearoyl-CoA desaturase (SCD) is an important regulatory enzyme of *de novo* lipogenesis, which catalyzes the biosynthesis of unsaturated fatty acids (Dobrzyn and Ntambi, 2004). Lipid has two-sided. An appropriate increase can produce a protective effect and an excessive increase can cause oxidative damage, lead to tissue lipid peroxidation, and finally cause lipotoxicity. Lipid homeostasis is crucial to prevent lipotoxicity. Our *in vivo* and *in vitro* experiments showed that SQLE, SCD and HMGCS1 increased significantly in the CaOx-induced renal injury models, suggesting the existence of lipid nephrotoxicity and progressive renal injury.

In addition, more and more evidence supports the lipid metabolism mediated by p53 tumor suppressor. The studies of Lacroix M et al. (Lacroix et al., 2021) suggest the importance of p53-SCD axis in lipid metabolism. The effect of p53 on Mevalonate Pathway was also suggested in the study of Freed-Pastor WA et al. (Freed-Pastor et al., 2012). The results of molecular docking not only suggested that EA might interact with SCD, SQLE, HMGCS1 protein directly, but also suggested that p53 transcription factor might regulate the above three targets. The results of immunoblotting imply the regulatory effect. Therefore, we speculate that EA may not only directly bind to SCD, SQLE, HMGCS1 and inhibit its expression, but also indirectly affect p53 and further affect the three downstream proteins.

In CaOx-induce renal injury, crystals precipitate in renal tubules and interact with renal tubular epithelial cells to induce oxidative stress and inflammation (Khan et al., 2021). Excessive oxidative stress and inflammation will not only increase the deposition and retention of oxalate crystals in tubular cells, but also lead to the development of fibrosis (Khan, 2014). Epithelial mesenchymal transition (EMT) is an important initial link of renal interstitial fibrosis and plays an important role in the repair of renal tissue injury (Kalluri and Neilson, 2003). In renal crystal induced renal injury, EMT occurs in renal tubular epithelial cells in the early stage of renal stone formation or crystal induced renal injury, and then triggers the process of renal fibrosis (Hu et al., 2015). Sun Y et al. (Sun et al., 2020) confirmed that the increase of renal cholesterol caused by abnormal cholesterol metabolism will increase oxidative stress injury. Kong YL et al. (Kong et al., 2020) confirmed that the excessive accumulation of cholesterol in cells may stimulate the increase of NLRP3 and induce inflammatory response. In addition, Accumulation of cholesterol can activate PI3K-Akt signaling pathway (Yue et al., 2014). Activated PI3K-Akt signaling pathway will aggravate renal inflammation and oxidative stress injury (Ang et al., 2015). Si YC et al. (Si et al., 2021) confirmed that inhibiting the activation of PI3K-Akt signaling pathway in oxalate crystallization mouse model can reduce crystalline kidney injury and inhibit the occurrence and development of EMT. In conclusion, we can speculate that abnormal cholesterol metabolism leads to the accumulation of cholesterol, which may increase the damage of oxidative stress, inflammation and fibrosis in the kidney.

**FIGURE 7:**
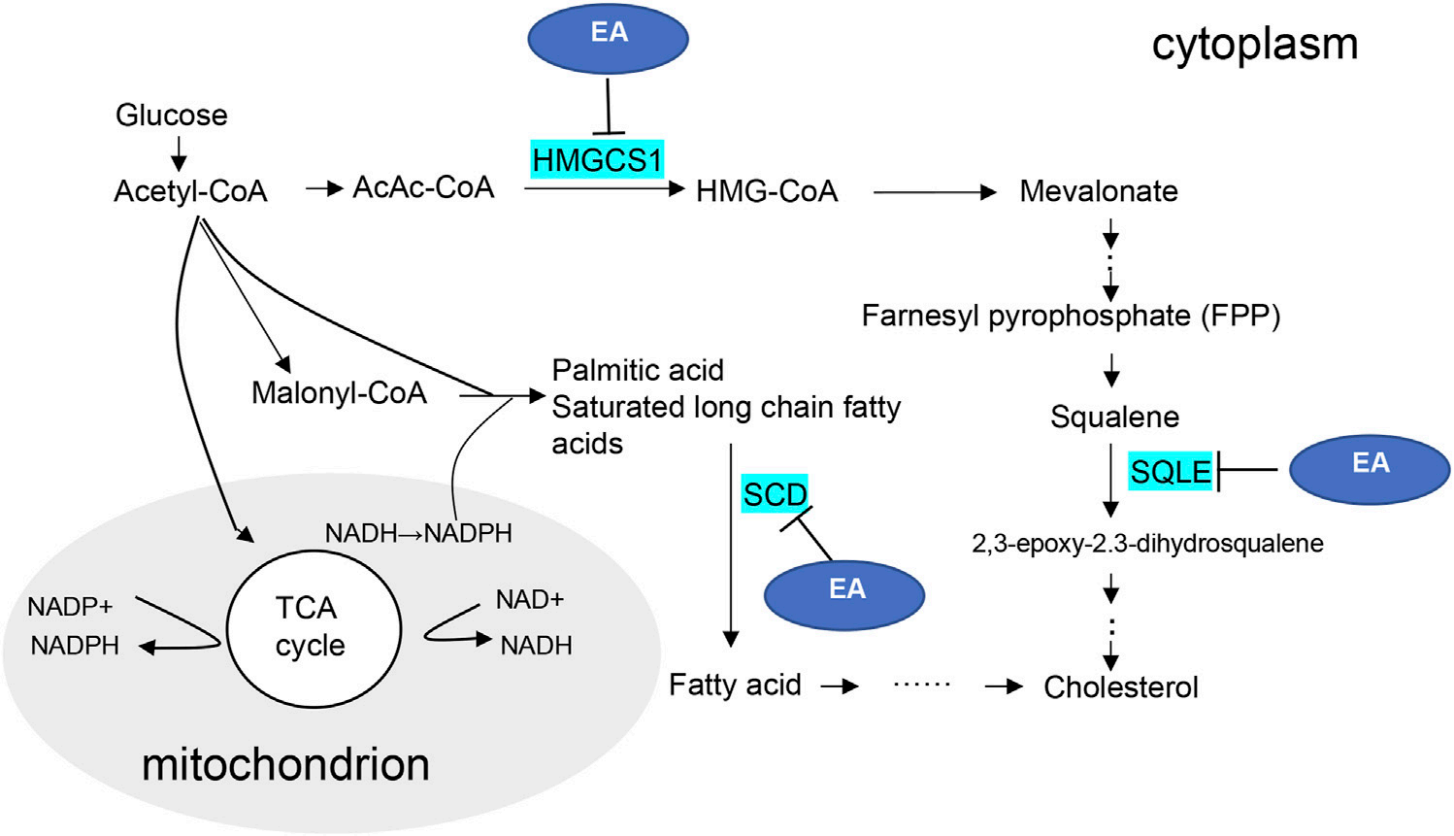
Diagram of the putative regulatory mechanisms of the ellagic acid.

EA is an active natural polyphenol ingredient with antibacterial, anti-inflammatory, hepatoprotective, anti-obesity, and anti-tumor effects (Chen et al., 2018). Furthermore, several studies have shown that EA can modulate lipid metabolism, which inhibits lipid accumulation by suppressing early adipogenic events and cell cycle arrest (Okla et al., 2015; Woo et al., 2015). Therefore, synthesize our experimental verification results, the possible mechanism diagram of EA regulating lipid metabolism in CaOx-induced renal injury was shown in Figure 7. Considering the molecular docking results, we speculate that EA may reduce the transformation of cholesterol in damaged HK-2 cells and protect cells in CaOx-induced renal injury by inhibiting the activities of HMGCS1, SCD, and SQLE.

## Data Availability

The datasets presented in this study can be found in online repositories. The names of the repository/repositories and accession number(s) can be found in the article/Supplementary Material.

## References

[B1] Abu-Ghanem Y. Kleinmann N. Winkler H. Z. Zilberman D. E. (2016). Nephrolithiasis in Israel: Epidemiological Characteristics of Return Patients in a Tertiary Care Center. Isr. Med. Assoc. J. 18, 725–728. 28457074

[B2] Ang L. Yuguang L. Liying W. Shuying Z. Liting X. Shumin W. (2015). Ergosterol Alleviates Kidney Injury in Streptozotocin-Induced Diabetic Mice. Evid. Based Complement. Altern. Med. 2015, 691594. 10.1155/2015/691594 PMC4664816 26664454

[B3] Bagalkotkar G. Sagineedu S. R. Saad M. S. Stanslas J. (2006). Phytochemicals from Phyllanthus Niruri Linn. And Their Pharmacological Properties: a Review. J. Pharm. Pharmacol. 58, 1559–1570. 10.1211/jpp.58.12.0001 17331318

[B4] Brown M. S. Goldstein J. L. (1997). The SREBP Pathway: Regulation of Cholesterol Metabolism by Proteolysis of a Membrane-Bound Transcription Factor. Cell. 89, 331–340. 10.1016/s0092-8674(00)80213-5 9150132

[B5] Chen P. Chen F. Zhou B. (2018). Antioxidative, Anti-inflammatory and Anti-apoptotic Effects of Ellagic Acid in Liver and Brain of Rats Treated by D-Galactose. Sci. Rep. 8, 1465. 10.1038/s41598-018-19732-0 29362375 PMC5780521

[B6] Dobrzyn A. Ntambi J. M. (2004). The Role of Stearoyl-CoA Desaturase in Body Weight Regulation. Trends Cardiovasc Med. 14, 77–81. 10.1016/j.tcm.2003.12.005 15030794

[B7] Evan A. P. (2010). Physiopathology and Etiology of Stone Formation in the Kidney and the Urinary Tract. Pediatr. Nephrol. 25, 831–841. 10.1007/s00467-009-1116-y 19198886 PMC2839518

[B8] Freed-Pastor W. A. Mizuno H. Zhao X. Langerød A. Moon S. H. Rodriguez-Barrueco R. (2012). Mutant P53 Disrupts Mammary Tissue Architecture via the Mevalonate Pathway. Cell. 148 (1-2), 244–258. 10.1016/j.cell.2011.12.017 22265415 PMC3511889

[B9] Freitas A. M. Schor N. Boim M. A. (2002). The Effect of Phyllanthus Niruri on Urinary Inhibitors of Calcium Oxalate Crystallization and Other Factors Associated with Renal Stone Formation. BJU Int. 89, 829–834. 10.1046/j.1464-410x.2002.02794.x 12010223

[B10] Gruenbacher G. Thurnher M. (2015). Mevalonate Metabolism in Cancer. Cancer Lett. 356, 192–196. 10.1016/j.canlet.2014.01.013 24467965

[B11] Hu H. Chen W. Ding J. Jia M. Yin J. Guo Z. (2015). Fasudil Prevents Calcium Oxalate Crystal Deposit and Renal Fibrogenesis in Glyoxylate-Induced Nephrolithic Mice. Exp. Mol. Pathol. 98 (2), 277–285. 10.1016/j.yexmp.2015.02.006 25697583

[B12] Huang M. Y. Chaturvedi L. S. Koul S. Koul H. K. (2005). Oxalate Stimulates IL-6 Production in HK-2 Cells, a Line of Human Renal Proximal Tubular Epithelial Cells. Kidney Int. 68, 497–503. 10.1111/j.1523-1755.2005.00427.x 16014026

[B14] Jantan I. Haque M. A. Ilangkovan M. Arshad L. (2019). An Insight into the Modulatory Effects and Mechanisms of Action of Phyllanthus Species and Their Bioactive Metabolites on the Immune System. Front. Pharmacol. 10, 878. 10.3389/fphar.2019.00878 31440162 PMC6693410

[B15] Kalluri R. Neilson E. G. (2003). Epithelial-mesenchymal Transition and its Implications for Fibrosis. J. Clin. Investig. 112 (12), 1776–1784. 10.1172/JCI20530 14679171 PMC297008

[B16] Kaur N. Kaur B. Sirhindi G. (2017). Phytochemistry and Pharmacology of Phyllanthus Niruri L.: A Review. Phytother. Res. 31, 980–1004. 10.1002/ptr.5825 28512988

[B17] Khan S. R. Canales B. K. Dominguez-Gutierrez P. R. (2021). Randall's Plaque and Calcium Oxalate Stone Formation: Role for Immunity and Inflammation. Nat. Rev. Nephrol. 17 (6), 417–433. 10.1038/s41581-020-00392-1 33514941

[B18] Khan S. R. Pearle M. S. Robertson W. G. Gambaro G. Canales B. K. Doizi S. (2016). Kidney Stones. Nat. Rev. Dis. Prim. 2, 16008. 10.1038/nrdp.2016.8 27188687 PMC5685519

[B19] Khan S. R. (2004). Role of Renal Epithelial Cells in the Initiation of Calcium Oxalate Stones. Nephron Exp. Nephrol. 98, e55–60. 10.1159/000080257 15499208

[B20] Khan S. R. (2014). Reactive Oxygen Species, Inflammation and Calcium Oxalate Nephrolithiasis. Transl. Androl. Urol. 3 (3), 256–276. 10.3978/j.issn.2223-4683.2014.06.04 25383321 PMC4220551

[B21] Kieley S. Dwivedi R. Monga M. (2008). Ayurvedic Medicine and Renal Calculi. J. Endourol. 22, 1613–1616. 10.1089/end.2008.0020 18620498

[B22] Kim H. J. Moradi H. Yuan J. Norris K. Vaziri N. D. (2009). Renal Mass Reduction Results in Accumulation of Lipids and Dysregulation of Lipid Regulatory Proteins in the Remnant Kidney. Am. J. Physiol. Ren. Physiol. 296, F1297–F1306. 10.1152/ajprenal.90761.2008 PMC2692452 19357177

[B23] Kim Y. J. Oh S. H. Ahn J. S. Yook J. M. Kim C. D. Park S. H. (2020). The Crucial Role of Xanthine Oxidase in CKD Progression Associated with Hypercholesterolemia. Int. J. Mol. Sci. 21, 7444. 10.3390/ijms21207444 33050202 PMC7589966

[B24] Kong Y. Feng W. Zhao X. Zhang P. Li S. Li Z. (2020). Statins Ameliorate Cholesterol-Induced Inflammation and Improve AQP2 Expression by Inhibiting NLRP3 Activation in the Kidney. Theranostics 10 (23), 10415–10433. 10.7150/thno.49603 32929357 PMC7482822

[B25] Krishnamurti G. V. Seshadri T. R. (1946). The Bitter Principle ofPhyllanthus Niruri. Proc. Indian Acad. Sci. Math. Sci.) 24, 357. 10.1007/BF03171072

[B26] Lacroix M. Linares L. K. Rueda-Rincon N. Bloch K. Di Michele M. De Blasio C. (2021). The Multifunctional Protein E4F1 Links P53 to Lipid Metabolism in Adipocytes. Nat. Commun. 12 (1), 7037. 10.1038/s41467-021-27307-3 34857760 PMC8639890

[B27] Laskowski R. A. Swindells M. B. (2011). LigPlot+: Multiple Ligand-Protein Interaction Diagrams for Drug Discovery. J. Chem. Inf. Model. 51, 2778–2786. 10.1021/ci200227u 21919503

[B28] Li X. R. Zhou W. Wei W. X. (2007). Chemical Components and Bioactivities of Phyllanthus Niruri L. Nat. Prod. Res. Dev. 19, 890. 10.16333/j.1001-6880.2007.05.040

[B29] Liu Q. Liang X. Liang M. Qin R. Qin F. Wang X. (2020). Ellagic Acid Ameliorates Renal Ischemic-Reperfusion Injury through NOX4/JAK/STAT Signaling Pathway. Inflammation 43, 298–309. 10.1007/s10753-019-01120-z 31768706

[B31] Neamatallah T. El-Shitany N. Abbas A. Eid B. G. Harakeh S. Ali S. (2020). Nano Ellagic Acid Counteracts Cisplatin-Induced Upregulation in OAT1 and OAT3: A Possible Nephroprotection Mechanism. Molecules. 25: 3031 10.3390/molecules25133031 32630784 PMC7411712

[B32] Nishiura J. L. Campos A. H. Boim M. A. Heilberg I. P. Schor N. (2004). Phyllanthus Niruri Normalizes Elevated Urinary Calcium Levels in Calcium Stone Forming (CSF) Patients. Urol. Res. 32, 362–366. 10.1007/s00240-004-0432-8 15221244

[B33] Okla M. Kang I. Kim D. M. Gourineni V. Shay N. Gu L. (2015). Ellagic Acid Modulates Lipid Accumulation in Primary Human Adipocytes and Human Hepatoma Huh7 Cells via Discrete Mechanisms. J. Nutr. Biochem. 26, 82–90. 10.1016/j.jnutbio.2014.09.010 25458530

[B34] Oner G. Cirrik S. (2009). The Nephrotoxicity Risk in Rats Subjected to Heavy Muscle Activity. J. Sports Sci. Med. 8, 481–488. 24150014 PMC3763296

[B35] Samuel W. Kutty R. K. Duncan T. Vijayasarathy C. Kuo B. C. Chapa K. M. (2014). Fenretinide Induces Ubiquitin-dependent Proteasomal Degradation of Stearoyl-CoA Desaturase in Human Retinal Pigment Epithelial Cells. J. Cell. Physiol. 229, 1028–1038. 10.1002/jcp.24527 24357007 PMC3999186

[B36] Scheid C. R. Cao L. C. Honeyman T. Jonassen J. A. (2004). How Elevated Oxalate Can Promote Kidney Stone Disease: Changes at the Surface and in the Cytosol of Renal Cells that Promote Crystal Adherence and Growth. Front. Biosci. 9, 797–808. 10.2741/1265 14766409

[B37] Si Y. Liu L. Cheng J. Zhao T. Zhou Q. Yu J. (2021). Oral Hydrogen-Rich Water Alleviates Oxalate-Induced Kidney Injury by Suppressing Oxidative Stress, Inflammation, and Fibrosis. Front. Med. 8, 713536. 10.3389/fmed.2021.713536 PMC8418222 34490303

[B38] Sun Y. Ge X. Li X. He J. Wei X. Du J. (2020). High-fat Diet Promotes Renal Injury by Inducing Oxidative Stress and Mitochondrial Dysfunction. Cell. Death Dis. 11 (10), 914. 10.1038/s41419-020-03122-4 33099578 PMC7585574

[B47] Taguchi K. Chen L. Usawachintachit M. Hamamoto S. Kang M. Sugino T. (2020). Fatty Acid-Binding Protein 4 Downregulation Drives Calcification in the Development of Kidney Stone Disease. Kidney Int. 97, 1042–1056. 10.1016/j.kint.2020.01.042 32247632

[B39] Thurnher M. Gruenbacher G. Nussbaumer O. (2013). Regulation of Mevalonate Metabolism in Cancer and Immune Cells. Biochim. Biophys. Acta 1831, 1009–1015. 10.1016/j.bbalip.2013.03.003 23524243

[B40] Trott O. Olson A. J. (2010). AutoDock Vina: Improving the Speed and Accuracy of Docking with a New Scoring Function, Efficient Optimization, and Multithreading. J. Comput. Chem. 31, 455–461. 10.1002/jcc.21334 19499576 PMC3041641

[B30] Wang B. Wei J. Huangfu Q. Gao F. Qin L. Zhong J. (2022). Identification of Resolvin D1 and Protectin D1 as Potential Therapeutic Agents for Treating Kidney Stones. Oxid. Med. Cell Longev. 2022, 4345037. 10.1155/2022/4345037 35251472 PMC8894018

[B41] Wang W. Fan J. Huang G. Li J. Zhu X. Tian Y. (2017). Prevalence of Kidney Stones in Mainland China: A Systematic Review. Sci. Rep. 7, 41630. 10.1038/srep41630 28139722 PMC5282506

[B42] Woo M. S. Choi H. S. Seo M. J. Jeon H. J. Lee B. Y. (2015). Ellagic Acid Suppresses Lipid Accumulation by Suppressing Early Adipogenic Events and Cell Cycle Arrest. Phytother. Res. 29, 398–406. 10.1002/ptr.5264 25462071

[B43] Yu Z. He Q. Xu G. (2020). Screening of Prognostic Factors in Early-Onset Breast Cancer. Technol. Cancer Res. Treat. 19, 1533033819893670. 10.1177/1533033819893670 32028860 PMC7011326

[B44] Yue S. Li J. Lee S. Y. Lee H. J. Shao T. Song B. (2014). Cholesteryl Ester Accumulation Induced by PTEN Loss and PI3K/AKT Activation Underlies Human Prostate Cancer Aggressiveness. Cell. Metab. 19, 393–406. 10.1016/j.cmet.2014.01.019 24606897 PMC3969850

[B45] Zager R. A. Burkhart K. M. Johnson A. C. Sacks B. M. (1999). Increased Proximal Tubular Cholesterol Content: Implications for Cell Injury and "acquired Cytoresistance". Kidney Int. 56, 1788–1797. 10.1046/j.1523-1755.1999.00745.x 10571787

[B46] Zager R. A. Johnson A. C. Becker K. (2011). Acute Unilateral Ischemic Renal Injury Induces Progressive Renal Inflammation, Lipid Accumulation, Histone Modification, and "End-Stage" Kidney Disease. Am. J. Physiol. Ren. Physiol. 301, F1334–F1345. 10.1152/ajprenal.00431.2011 PMC3233867 21921025

