# Corrigendum: *Phyllanthus* niruri L. exerts protective effects against the calcium oxalate-induced renal injury via ellgic acid

**DOI:** 10.3389/fphar.2023.1207268

**Published:** 2023-06-09

**Authors:** Mao-Ting Li, Lu-Lu Liu, Qi Zhou, Lin-Xi Huang, Yu-Xuan Shi, Jie-Bin Hou, Hong-Tao Lu, Bing Yu, Wei Chen, Zhi-Yong Guo

**Affiliations:** ^1^ Changhai Hospital, Naval Medical University, Shanghai, China; ^2^ Department of Nephrology, the Second Medical Centre, Chinese PLA General Hospital, Beijing, China; ^3^ Department of Naval Medicine, Naval Medical University, Shanghai, China; ^4^ Department of Cell Biology, Center for Stem Cell and Medicine, Navy Medical University, Shanghai, China

**Keywords:** *Phyllanthus* niruri L., calcium oxalate-induced renal injury, network pharmacology, ellagic acid, lipid nephrotoxicity

In the published article, there were errors in Figure 4A and Figure 5D as published In Figure 4A, the concentration unit of EA in the column chart is marked as “mM”, while the correct concentration unit in the text is “μM”. In Figure 5D (Con/SQLE) panels, due an error had been made in the labelling of the original images, the immunohistochemical images of the Con/SQLE groups were incorrectly selected. We revised the semi-quantitative histograms of the positive expression area of HMGCS1, SCD and SQLE in Figure 5D in order to present the results more clearly. The corrected Figure 4 and Figure 5 and their caption appear below.

**FIGURE 4:**
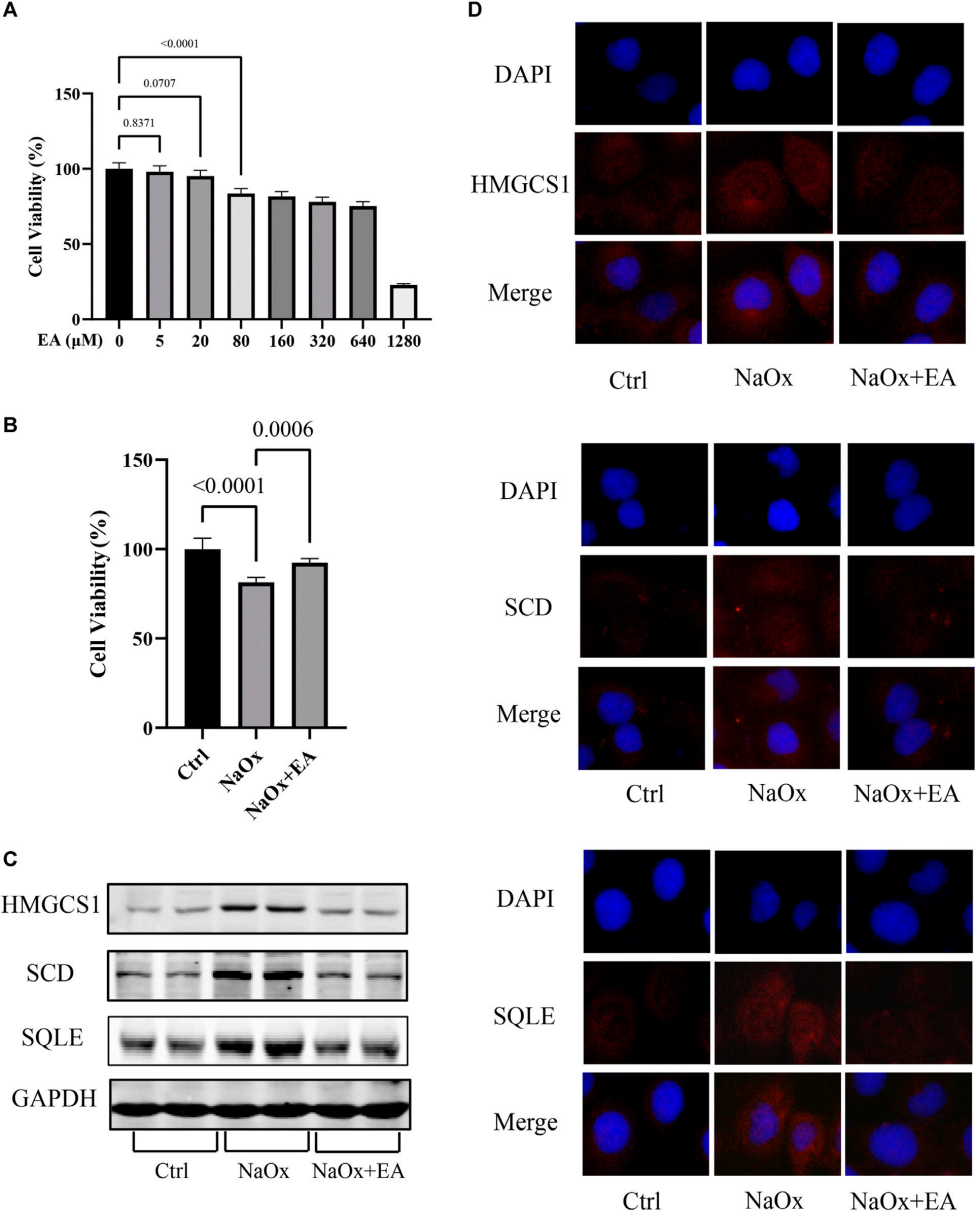
Ellagic acid protects NaOx-induced injury in cells and reduces the expression of SQLE, HMGCS1, and SCD **(A)** The cytotoxicity of ellagic acid on HK-2 cells determined by the CCK8 assay **(B)** The cytotoxicity of ellagic acid on NaOx treated HK-2 cells determined by the CCK8 assay **(C)** The expression of SQLE, HMGCS1, and SCD by Western blot in the control group (Ctrl), sodium oxalate group (NaOx), and ellagic acid treatment group (NaOx + EA) **(D)** Cells were analyzed by immunocytochemistry (100X) in the control group (Ctrl), sodium oxalate group (NaOx), and ellagic acid treatment group (NaOx + EA).

**FIGURE 5:**
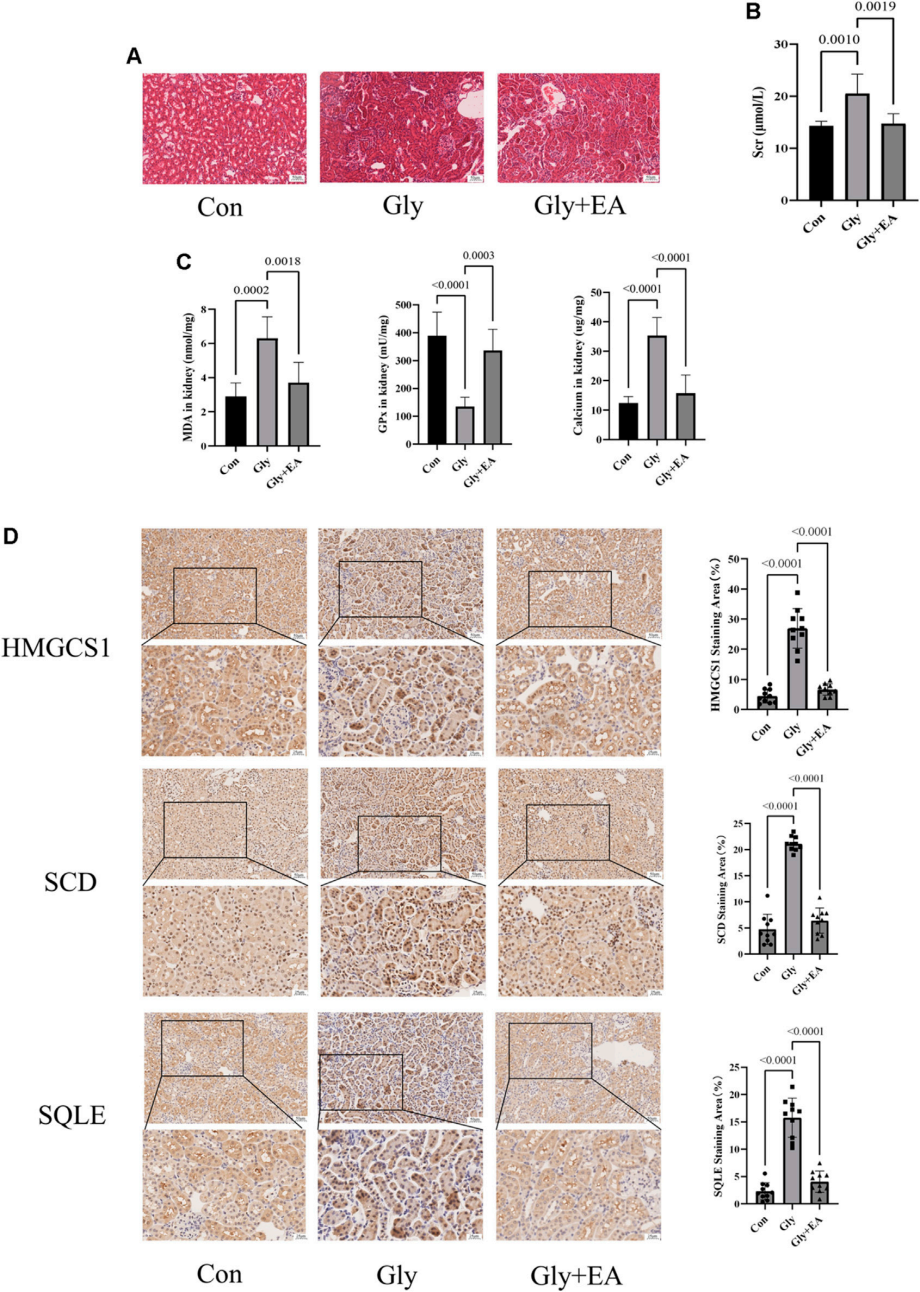
Ellagic acid protects calcium oxalate-induced renal injury in mice and reduces the expression of SQLE, HMGCS1, and SCD **(A)** Representative light microscopy images of hematoxylin and eosin staining of kidneys from the control group (Con), glyoxylate-induced CaOx group (Gly), and ellagic acid treatment group (Gly + EA) (magnification, ×200; scale bar = 50 μm) **(B)** Serum creatinine level in control group (Con), glyoxylate-induced CaOx group (Gly), and ellagic acid treatment group (Gly + EA) **(C)** The GPx activities, MDA, and total calcium content in control group (Con), glyoxylate-induced CaOx group (Gly), and ellagic acid treatment group (Gly + EA) **(D)** Expression of SCD, HMGCS1, and SQLE were analyzed by immunohistochemistry (IHC) (left) Representative light microscopy images of IHC staining of kidney of mice (magnification, ×200; scale bar = 50 μm (up) and 25 μm (down)) (right) Semi-quantitative score of SCD, HMGCS1, and SQLE.

The authors apologize for this error and state that this does not change the scientific conclusions of the article in any way. The original article has been updated.

